# The bRPS6-Family Protein RFC3 Prevents Interference by the Splicing Factor CFM3b during Plastid rRNA Biogenesis in *Arabidopsis thaliana*

**DOI:** 10.3390/plants9030328

**Published:** 2020-03-04

**Authors:** Yumi Nagashima, Katsutomo Ohshiro, Akiyasu Iwase, Miyuki T. Nakata, Shugo Maekawa, Gorou Horiguchi

**Affiliations:** 1 Department of Life Science, College of Science, Rikkyo University, Toshima, Tokyo 171-8501, Japan; 18ld007d@rikkyo.ac.jp (Y.N.); katsutomo0204@gmail.com (K.O.); iws.office.akys@gmail.com (A.I.); maeshu@rikkyo.ac.jp (S.M.); 2 Research Center for Life Science, College of Science, Rikkyo University, Toshima, Tokyo 171-8501, Japan; miyuki-t-nakata@bs.naist.jp; 3 Graduate School of Science and Technology, Nara Institute of Science and Technology, Ikoma, Nara 630-0192, Japan

**Keywords:** *Arabidopsis*, CFM3b, lateral root, plastid, RFC3, ribosome, rRNA biogenesis, *sprt2*

## Abstract

Plastid ribosome biogenesis is important for plant growth and development. REGULATOR OF FATTY ACID COMPOSITION3 (RFC3) is a member of the bacterial ribosomal protein S6 family and is important for lateral root development. *rfc3-2* dramatically reduces the plastid rRNA level and produces lateral roots that lack stem cells. In this study, we isolated a *suppressor of rfc three2* (*sprt2*) mutant that enabled recovery of most *rfc3* mutant phenotypes, including abnormal primary and lateral root development and reduced plastid rRNA level. Northern blotting showed that immature and mature plastid rRNA levels were reduced, with the exception of an early 23S rRNA intermediate, in *rfc3-2* mutants. These changes were recovered in *rfc3-2 sprt2-1* mutants, but a second defect in the processing of 16S rRNA appeared in this line. The results suggest that *rfc3* mutants may be defective in at least two steps of plastid rRNA processing, one of which is specifically affected by the *sprt2-1* mutation. *sprt2-1* mutants had a mutation in *CRM FAMILY MEMBER 3b* (*CFM3b*), which encodes a plastid-localized splicing factor. A bimolecular fluorescence complementation (BiFC) assay suggested that RFC3 and SPRT2/CFM3b interact with each other in plastids. These results suggest that RFC3 suppresses the nonspecific action of SPRT2/CFM3b and improves the accuracy of plastid rRNA processing.

## 1. Introduction

During plant evolution, endosymbiotic ancestral cyanobacteria and α-proteobacteria were engulfed by host eukaryotic cells and evolved into plastids and mitochondria, respectively [1,2]. Thus, plants have three different types of ribosomes classified as prokaryotic (mitochondrial and plastidial ribosomes) and eukaryotic (cytosolic ribosomes). The 70S plastid ribosome is composed of a 50S subunit and a 30S subunit. The 50S subunit includes approximately 30 ribosomal proteins (RPs) as well as 23S, 5S, and 4.5S rRNAs, while the 30S subunit includes approximately 20 RPs and 16S rRNA [3,4,5,6]. Among the components of the plastid translation system, RPs are encoded in the plastid and nuclear genomes [7], while all tRNAs and rRNAs are encoded by the plastid genome [8]. The rRNAs are transcribed from single operon containing *rrn16*, *trnI*, *trnA*, *rrn23*, *rrn4.5*, *rrn5*, and *trnR*, in this order; these rRNAs undergo processing by endonucleolytic cleavage, exonucleolytic trimming, nucleotide modifications, and ternary structure folding [7,9,10,11,12,13]. Primary *rrn* transcripts are cleaved into three fragments, each containing pre-16S, pre-23S-4.5S, and pre-5S rRNAs. The pre-23S-4.5S rRNA is cleaved into pre-23S and pre-4.5S rRNAs. These monocistronic fragments are trimmed to yield mature rRNAs. Concurrently, RPs bind immature rRNAs to assist their folding and produce mature 30S and 50S subunits. These processes are assisted by multiple ribosome biogenesis factors [7]. The mature 23S rRNA is further cleaved into three fragments at sites known as hidden breaks [14]. Thus far, many plastidial ribosome biogenesis factors have been identified, but details of ribosome biogenesis in plastids remains unclear.

RNA splicing also plays an important role in successful gene expression in plastids. The plastid genome contains groups I and II introns; group II introns are divided into subclasses IIA and IIB [15]. These introns do not exhibit autocatalytic activity and require proteinaceous factors [16]. Biochemical and molecular genetic analyses in *Arabidopsis thaliana* (hereafter, *Arabidopsis*), maize, and rice have identified many factors involved in RNA splicing in plastids [17]. Among them, chloroplast RNA splicing and ribosome maturation (CRM) domain proteins have been extensively characterized [18]. The CRM domain has a conserved GxxG motif and RNA binding activity has been demonstrated in some CRM domain proteins [18]. The only CRM domain protein in *Escherichia coli*, YhbY, has a single CRM domain; this protein binds to pre-50S ribosomes and promotes their maturation [18]. A recent study discovered that YhbY also plays a role in 30S subunit biogenesis [19]. CRM domain proteins in plants diversified during evolution and are classified into four subfamilies (CHLOROPLAST RNA SPLICING1 [CRS1] and CRS2-ASSOCATED FACTOR [CAF], subfamilies 3 and 4), according to the number of CRM domains and the presence or absence of additional motifs [18]. CAF1 and CAF2 contain two CRM domains and are involved in the splicing of group IIB introns in plastids [20]. They recognize different subsets of group IIB introns and form heterodimers with CRS2, an RNA splicing factor related to peptidyl-tRNA hydrolase [20,21]. The CRS1 subfamily members have three or four CRM domains and include CFM2 and CFM3 [18]. Thus far, all known CRS1 subfamily members localize to plastids and promote RNA splicing [16,18,22,23,24]. CRS1 specifically binds the group IIA intron of *atpF* and promotes its folding into the tertiary structure necessary for its catalytic activity [16]. On the other hand, CFM2 is involved in the splicing of *trnL* (a group I intron), as well as *ndhA* and *yfc3* intron1 (group IIB introns) [24]. In maize, CFM3 also promotes splicing of a different subset of group IIB introns (*ndhB*, *petB*, *petD*, *rpl16*, *rps16*, and *trnG*), relative to those recognized by CFM2 [23]. In *Arabidopsis*, CFM3 has two paralogs, CFM3a and CFM3b, both of which are localized in plastids; these paralogs presumably play redundant roles in group IIB intron splicing, in which CFM3a has a major role [23]. Two other CRM domain proteins, CFM9 and mitochondria CAF-like splicing factor 1 (mCSF1), localize to mitochondria and are involved in splicing of mitochondrial transcripts [25,26].

Although most CRM domain proteins in plants play crucial roles in the splicing of organellar groups I and II introns, a few have different roles. For example, CFM4 localizes to plastids and is suspected to be involved in 16S and 4.5S processing via its RNA chaperone activity [27]. Interestingly, CFM3 in maize and CFM3a (but not CFM3b) in *Arabidopsis* are also targeted to mitochondria and play roles in mitochondrial ribosome small subunit maturation [23].

Plastid development and plastid ribosome biogenesis are coordinated with the roles of plastids in different cell types, tissues, and organs, as well as with changes in environmental conditions. However, apart from chloroplast biogenesis in response to light [28,29,30], little is known regarding the mechanisms underlying this coordination in non-photosynthetic organs [31,32]. We previously identified REGULATOR OF FATTY ACID COMPOSITION3 (RFC3) as a member of the bacterial ribosomal protein S6 (bRPS6) family and found that it is important for lateral root (LR) development [33,34,35]. The plastid ribosome contains a *bona fide* RPS6 encoded by the nuclear *PLASTID RIBOSOMAL PROTEIN S6* (*PRPS6*) gene, which originates from the cyanobacterial ancestor [34,35]. Although plastid RPs retain considerable homology with their bacterial RP counterparts [7], RCF3 is distantly related to PRPS6 and is rather placed in the same clade as β/γ proteobacteria [35]. Loss-of-function of RFC3 results in striking reductions in the 23S and 16S rRNA levels in roots [35]. Therefore, RFC3 may be involved in the biogenesis or stability of plastid rRNAs.

*rfc3* mutants show an interesting developmental defect; they fail to complete patterning of the root apical meristem (RAM) in LRs [33,34]. In *Arabidopsis*, LR formation is divided into stages I to VII and initiate from pericycle cells associated with protoxylem poles [36]. During the course of LR development, specific modules of AUXIN RESPONSE FACTOR (ARF) transcription factors and AUXIN/INDOLE ACETIC ACID (AUX/IAA) repressor proteins, as well as other downstream transcription factors, specify LR primordium (LRP), founder cells and promote LR formation [37,38,39]. In parallel, three PLETHORA transcription family members—PLT3, PLT5, and PLT7—contribute to the establishment of LRPs [40]. During LR development, quiescent center (QC) is established by the action of a transcription factor complex comprising PLT, SCARECROW (SCR), and several members of TEOSINTE BRANCHED1/CYCLOIDEA/PCF (TCP) through activation of *WUSCHEL-RELATED HOMEOBOX5* (*WOX5*) expression [41,42]. While these studies have advanced our understanding of the molecular and cellular processes of LR development, another process may be required for appropriate LR development as described below.

The *rfc3* mutant produces nodule-like LRs that lack a QC and a functional RAM [35]. Furthermore, the expression levels of *PLT3* and *PLT7* are higher in roots of *rfc3* mutants than in the wild type (WT) [35]. Because WT plants treated with translation inhibitors for bacteria-type ribosomes develop *rfc3*-like abnormal LRs, plastid ribosomes likely play important roles in LR development [35]. To better understand the relationships among RFC3, plastid ribosome, and development of primary root (PR) and LR, we carried out suppressor screening using *rfc3-2* mutants. We recovered *suppressor of rfc three* (*sprt*) mutants in which both abnormal PR and LR development and the reduced plastid rRNA level were rescued. We then identified the causal gene of the *sprt2* mutation as *CFM3b* and revealed interactions between RFC3 and CFM3b in plastids. In the context of these findings, we discuss the potential, yet unexpected, link between rRNA processing and splicing factors in plastids.

## 2. Results

### 2.1. Identification of Sprt Mutants

**Figure 1:**
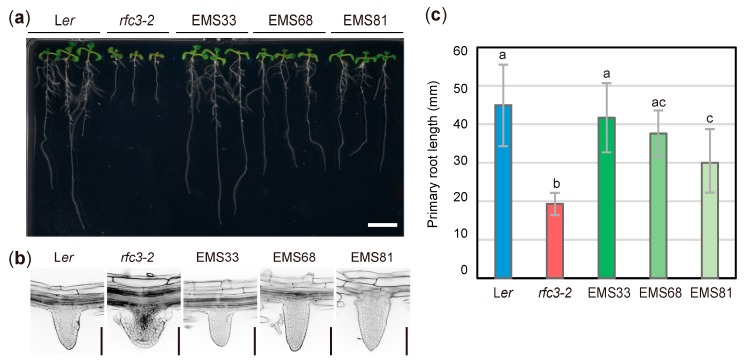
Isolation of *rfc3* suppressor mutants. (**a**) Plants grown on 1/2 MS medium containing 3% sucrose for 11 d. Bar = 1 cm. (**b**) Lateral root (LR) phenotypes observed by modified pseudo-Schiff-propidium iodide (mPS-PI) staining. Bars = 100 μm. (**c**) Primary root (PR) length. Data are means ± SDs (n ≥ 11). Statistical analysis was carried out by one-way ANOVA with Tukey HSD test (*p* < 0.05). Data without significant differences are labeled with the same letters. EMS33, EMS68, and EMS81 correspond to *rfc3-2 sprt1-1*, *rfc3-2 sprt2-1*, and *rfc3-2 sprt3-1*, respectively.

*rfc3-2* mutants were isolated from the Landsberg *erecta* (L*er*) background and produced short PRs with abnormal nodule-like LRs (Figure 1) [33,34]. Three suppressors (EMS33, EMS68, EMS81) of *rfc3* with elongated PRs and normal LRs were identified by visual inspection from EMS-mutagenized M2 populations (Figure 1a,c). EMS33 showed near full recovery of PR length compared to WT roots, while EMS81 had the shortest PRs among the three suppressors. EMS68 had an intermediate PR length (Figure 1c). The three suppressors produced LRs with a well-organized RAM, compared to the disorganized RAM in *rfc3-2* mutants (Figure 1b). The PR and LR phenotypes in F1 progenies among the three suppressors and *rfc3-2* mutants indicated that the second-site mutations in EMS33, EMS68, and EMS81 behaved as semi-dominant, recessive, and dominant alleles, respectively, when focused on the LR phenotype (Figure S1). Due to the semi-dominant and dominant behaviors of mutations in EMS 33 and EMS 81, allelism tests among the three suppressors were impractical (Figure S1). We thus tentatively assigned mutated loci in EMS33, EMS68, and EMS81 as *sprt1-1*, *sprt2-1*, and *sprt3-1*, respectively. We use the term “*sprt*s” to refer to these mutants collectively.

### 2.2. Most rfc3 Mutant Phenotypes were Recovered by the sprt2-1 Mutation

**Figure 2:**
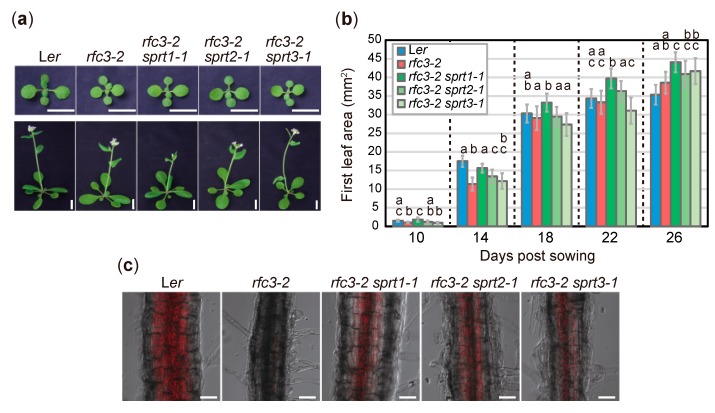
Shoot phenotypes and differentiation potentials of chloroplasts in *rfc3* and *rfc3 sprt*s. (**a**) Shoot phenotypes. Plants grown for 14 d (upper row) or 26 d (lower row) are shown. Bars = 1 cm. (**b**) Time-course analysis of first leaf area. Data are means ± SDs (n ≥ 12). For each group of samples harvested at different days, statistical analysis was carried out separately by one-way ANOVA with Tukey HSD test (*p* < 0.05). Data without significant differences are labeled with the same letters. (**c**) Differentiation of chloroplasts in the mature region of PRs. Chlorophyll autofluorescence (red) was merged with bright-field images. Bars = 50 μm.

To characterize the extent to which *sprt*s mutations suppressed *rfc3* mutant phenotypes, we examined several developmental, cellular, and gene expression phenotypes. *rfc3-2* mutants grew slowly and formed pale green leaves compared to WT plants [35]. A time-course analysis of leaf growth showed that in the early stages, *rfc3-2*, *rfc3-2 sprt2-1*, and *rfc3-2 sprt3-1* mutants formed smaller first leaves than WT plants, whereas the *rfc3-2 sprt1-1* mutants recovered this defect (Figure 2a,b). At the mature stage, however, neither *rfc3-2* nor *rfc3-2 sprt*s mutants produced smaller first leaves than WT leaves (Figure 2a,b). In WT but not *rfc3-2* roots, chlorophyll accumulated in plastids after prolonged exposure to light (Figure 2c). On the other hand, all *rfc3-2 sprt*s mutants re-accumulated chlorophyll in roots as determined by chlorophyll autofluorescence, albeit to a lesser extent than WT roots (Figure 2c).

**Figure 3:**
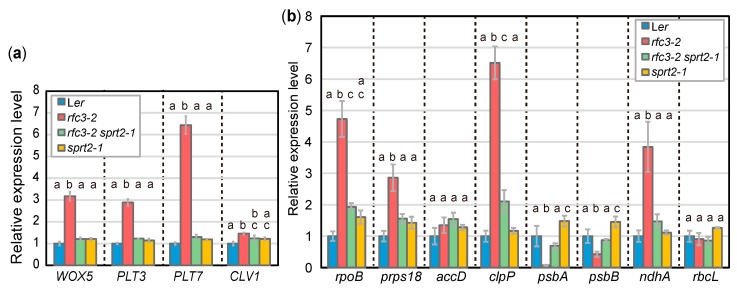
RNA expression of nuclear- and plastid-encoded genes in roots as determined by RT-qPCR. (**a**) Relative expression levels of nuclear-encoded genes normalized by *ACT2* expression level. (**b**) Relative expression levels of plastid-encoded genes normalized by 18S rRNA level. Data are means ± SDs. (n = 3). Statistical analysis was carried out by one-way ANOVA with Tukey HSD test for each mRNA species (*p* < 0.05). Data without significant differences are labeled with the same letters.

Next, we selected *rfc3-2 sprt2-1* mutants to further characterize gene expression phenotypes in roots by RT-qPCR, since we succeeded identification of the *sprt2-1* mutation point as described in a later part of this study (Figure 7). The identification of *sprt2-1* mutation site also allowed isolation of *sprt2-1* single mutants and they were included in some of following experiments. Further characterization of *rfc3-2 sprt1-1*, *rfc3-2 sprt3-1*, and corresponding single mutants will be reported elsewhere. The RNA expression levels of stem-cell regulatory genes, such as *WOX5*, *PLT3*, *PLT7*, and *CLAVATA1* (*CLV1*) [43], were increased in roots of *rfc3-2* mutants, compared to WT plants (Figure 3a) [35]. In *rfc3-2 sprt2-1* mutants, these genes were expressed at levels nearly identical to those in WT plants (Figure 3a). We also investigated the expression levels of several plastid-encoded genes in roots. Expression levels of *psbA* and *psbB* significantly decreased, while expression levels of *rpoB*, *prps18*, *clpP*, and *ndhA* significantly increased in *rfc3-2* mutants (Figure 3b). In *rfc3-2 sprt2-1* mutants, *psbA* and *psbB* were expressed at levels similar to those in WT plants (Figure 3b). In addition, *rpoB*, *prps18*, *clpP*, and *ndhA* expression levels were only slightly higher in *rfc3-2 sprt2-1* mutants than in WT plants (Figure 3b). The expression levels of *accD* and *rbcL* did not show clear differences among the genotypes examined (Figure 3b). The expression levels of the above-mentioned nuclear and plastid RNAs in *sprt2-1* single mutants were similar to those in WT plants (Figure 3a,b).

**Figure 4:**
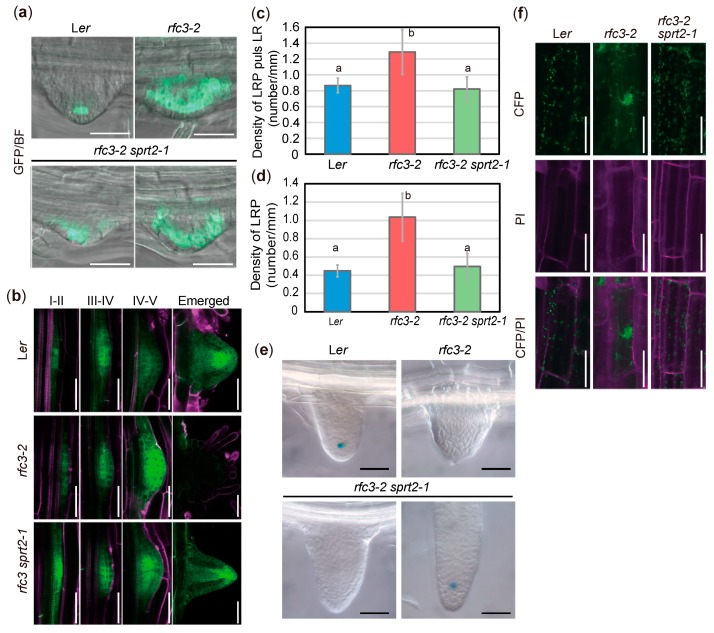
Reporter analyses of *rfc3-2* and *rfc3-2 sprt2-1* mutants. (**a**) p*WOX5*::*GFP* patterns (green) merged with bright-field (BF) images in LR primordia (LRPs). Bars = 100 μm. (**b**) p*PLT3*::*CFP* patterns in LRPs at different stages. p*PLT3*::*CYAN FLUORESCENT PROTEIN* (CFP) fluorescence is shown in green; roots were stained with propidium iodide (PI, magenta). Bars = 50 μm. (**c**) Density of CFP-positive LRPs plus LRs in PR of p*PLT3*::*CFP* lines at 7 days post sowing (dps). (**d**) Density of CFP-positive LRPs in PR of p*PLT3*::*CFP* lines at 7 dps. In (**c**) and (**d**), statistical analysis was carried out by one-way ANOVA with Tukey HSD test (*p* < 0.05, n = 10, means ± SDs). Data without significant differences are labeled with the same letters. (**e**) *QC25* expression patterns in emerged LRs. Bars = 50 μm. Lower left and right panels show a young and an elongated LRs, respectively. (**f**) Intracellular distribution of plastids observed in p*35S*::*RecATP-CFP* lines. CFP fluorescence is shown in green; roots were stained with PI (magenta). Bars = 50 μm.

Next, we examined the spatial expression patterns of LR regulatory genes during LR development. Expression of p*WOX5*::*GREEN FLUORESCENT PROTEIN* (*GFP*) was QC-specific in WT plants, whereas it was expressed throughout the LRPs in *rfc3-2* mutants (Figure 4a) [35]. In *rfc3-2 sprt2-1* mutants, the expression of p*WOX5*::*GFP* was expanded, but partially localized to QCs of LRPs (Figure 4a). Furthermore, in nearly all mature LRs of *rfc3-2 sprt2-1* mutant, p*WOX5*::*GFP* was expressed specifically in QC cells (Figure S2). p*PLT3*::*CYAN FLUORESCENT PROTEIN* (*CFP*) was expressed in early LRPs and mainly in the stem-cell niche in mature LRs (Figure 4b) [44]. In *rfc3-2* mutants, the p*PLT3*::*CFP* expression pattern in the early LRPs was relatively normal, but may be upregulated during stages IV–V and disappeared in completely differentiated abnormal LRs (Figure 4b). This result alone is difficult to explain the enhanced expression of *PLT3* in *rfc3-2* mutants (Figure 3a). To solve this inconsistency, we focused on LR and LRP density. p*PLT3*::*CFP* is active throughout LRP development and in mature LRs in the WT plants and *rfc3-2 sprt2-1* mutants (Figure 4b). On the other hand, mature LRs in *rfc3-2* lost the p*PLT3*::*CFP* expression (Figure 4b). Therefore, we counted the numbers of CFP*-*positive LRs and LRPs, respectively, and their density were determined by dividing with the PR length of individual plants. Concerning LRP, *rfc3-2* had about 2-fold higher density than WT plants and *rfc3-2 sprt2-1* mutants (Figure 4d). When densities of LRP plus LR were compared, *rfc3-2* mutants had only about 1.5-fold higher density than WT plants, reflecting disappearance of p*PLT3*::*CFP* expression in fully differentiated abnormal LRs in *rfc3-2* mutants. (Figure 4c,d). Therefore, while the expression of *WOX5* increased in *rfc3-2* mutants due to a combined effect of ectopic expression in LRPs and an increased density of LRPs, the increased expression of *PLT3* in *rfc3-2* mutants was mainly due to the increased LRP density in *rfc3-2* mutants.

We next examined the identities of QC in LRPs because *rfc3-2* mutants failed to establish LRs beyond stage VI, had no structurally discernable QC, and were devoid of *QC25*::*GUS* expression (Figure 4e) [35]. Expression of *QC25*::*GUS* was undetectable in young LRs of *rfc3-2* and *rfc3-2 sprt2-1* but expressed in sufficiently elongated *rfc3-2 sprt2-1* LRs (Figure 4e). Under our experimental conditions, *QC184*::*GUS* was expressed in QC, as well as the mature parts of WT roots (Figure S3). In fully differentiated *rfc3-2* mutant LRs, the strong GUS signal likely reflected expression of *QC184*::*GUS* in mature roots, rather than expression caused by ectopic QC identity (Figure S3). In elongating LRs of rfc3-2 sprt2-1 mutants, QC184::GUS expression was not observed (Figure S3). Therefore, QC identity and function were progressively but incompletely restored in rfc3-2 sprt2-1 mutants. rfc3-2 mutants also exhibited alterations in the intracellular distributions of plastids in roots. When root plastids were visualized using CFP fused to the RecA transit peptide, plastid distribution was partially restored from a clustered pattern to a scattered pattern in rfc3-2 sprt2-1 mutants (Figure 4f).

### 2.3. rfc3-2 sprt2-1 Mutants Exhibit Restoration of Plastid rRNA Accumulation

**Figure 5:**
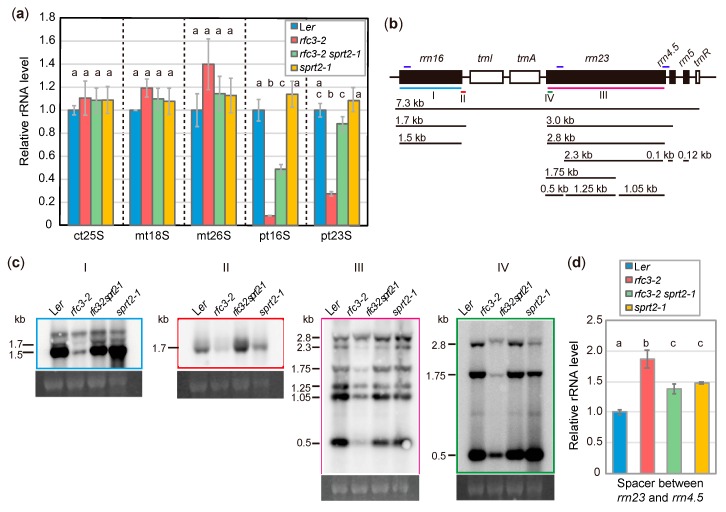
Analysis of rRNA accumulation and processing in *rfc3-2* and *rfc3-2 sprt2-1* mutants. (**a**) Cytosolic and organellar rRNA levels determined by RT-qPCR and normalized by 18S rRNA level. ct, mt, and pt indicate cytosolic, mitochondrial, and plastid rRNAs, respectively. Data are means ± SDs (n = 3). (**b**) Diagram of the plastid rRNA operon and the processing pattern. The position of probes for Northern analysis are indicated by colored bars below the diagram. The regions amplified by RT-qPCR are indicated by dark blue bars above the diagram. (**c**) Northern analysis of plastidial rRNA processing in roots. An ethidium bromide-stained image of 18S rRNA is shown below each blot as a loading control. (**d**) Levels of spacer region between *rrn23* and *rrn4.5* determined by RT-qPCR. Data are means ± SDs (n = 3). In (**a**) and (**d**), statistical analyses were carried out by one-way ANOVA with Tukey HSD test (*p* < 0.05). Data without significant differences are labeled with the same letters.

The recovery of most *rfc3* mutant phenotypes in *rfc3-2 sprt2-1* mutants suggested that the *sprt2-1* mutation affected a process associated with the molecular function of RFC3. Thus, we next analyzed plastid rRNAs and their precursors in roots of *rfc3-2 sprt2-1* mutants. The levels of 16S and 23S rRNAs in *rfc3-2* mutant roots were reduced to approximately 10% and 30% of the levels in WT plants, respectively (Figure 5a) [35]. In contrast, cytosolic and mitochondrial rRNA levels were similar in all genotypes examined (Figure 5a). In *rfc3-2 sprt2-1* mutant roots, the respective 16S and 23S rRNA levels recovered to approximately half and nearly identical to those in WT roots (Figure 5a). The 16S and 23S rRNA levels were normal in *srpt2-1* single mutants (Figure 5a). This finding suggested that the *sprt2-1* mutation increased the 16S and 23S rRNA levels specifically in the *rfc3* mutant background. 

We next examined rRNA processing by Northern blotting. An overview of rRNA processing in plastids is shown in Figure 5b. When a probe covering the entire 16S rRNA was used, the amount of 16S rRNA was found to be greatly reduced in *rfc3-2* mutants (Figure 5c). Similarly, a probe specific to the 3′ region of the 1.7-kb 16S rRNA precursor revealed significant reduction in the level of this precursor in *rfc3-2* mutants (Figure 5c). In contrast, the mature 16S rRNA level was partially recovered in *rfc3-2 sprt2-1* mutants, but overaccumulation of the 1.7-kb 16S rRNA precursor was observed (Figure 5c). The 3.0-kb precursor of 23S rRNA is cleaved into a 2.8-kb 23S rRNA precursor and pre-4.5S rRNA, and then the 2.8-kb 23S rRNA is cleaved into three fragments (1.25, 1.05, and 0.5 kb) (Figure 5b). When a probe covering the entire 23S rRNA was used, these three fragments were less abundant in *rfc3-2* mutants, compared to WT plants (Figure 5c). Two 23S rRNA species were cut at one hidden break (1.75 and 2.3 kb), while uncut 23S rRNA was 2.8 kb. (Figure 5b); 1.75-kb and 2.3-kb 23S rRNA species were less abundant in *rfc3-2* mutants than in WT plants, but were recovered to WT levels in *rfc3-2 sprt2-1* mutants (Figure 5c). An exception was the longest RNA species detectable using the 23S rRNA probe, which is likely the 2.8-kb 23S rRNA and/or 3.0-kb precursors, as it was also detectable by a probe for the 5′ region of 23S rRNA (Figure 5b,c). The levels of these precursors seem to be less affected in *rfc3-*2 mutants when the levels of these and shorter 23S rRNA species were compared to those in WT plants (Figure 5c). To distinguish 3.0-kb precursor from 2.8-kb 23S rRNA, we carried out RT-qPCR using a primer pair that amplifies cDNA corresponding to a region from the 3′ end of 23S rRNA to the spacer region of that is found in the 3.0-kb 23S-4.5S rRNA and longer (7.3 kb) precursors. As a result, we found that *rfc3-2* mutants had about 1.9-fold higher level these rRNA precursors when compared to WT plants (Figure 5d), suggesting that either one or both of the 3.0-kb 23S-4.5S rRNA precursors and the 7.3-kb precursors over accumulated in *rfc3-2* mutants. On the other hand, *rfc3-2 sprt2-1* mutants showed an intermediate level of these precursor*s* (Figure 5d). Therefore, in *rfc3-2 sprt2-1* mutants, the abnormal rRNA processing of *rfc3-2* mutants was largely restored. In contrast, *sprt2-1* mutants did not show strong defects in the processing pattern (Figure 5c,d).

### 2.4. Effect of the sprt2-1 Mutation on Plastid Ribosome Biogenesis

**Figure 6:**
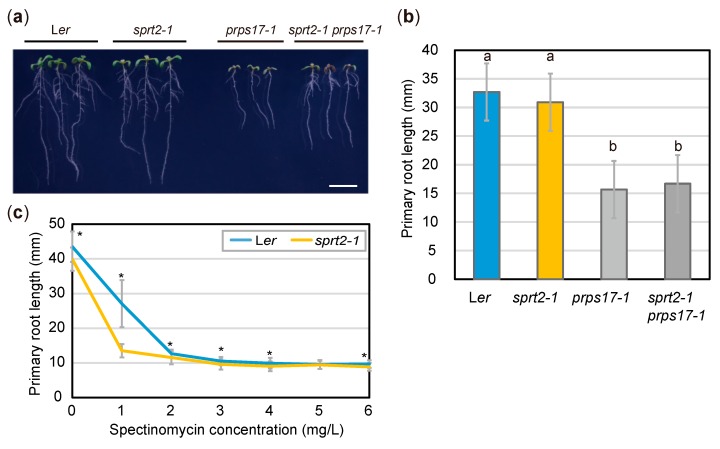
Effects of the *sprt2-1* mutation on abnormal ribosome biogenesis. (**a**) Plants grown on 1/2 MS medium with 3% sucrose for 8d. Bar = 1 cm. (**b**) PR lengths of seedlings at 8 dps. Data are means ± SDs (n = 24). Statistical analysis was carried out by one-way ANOVA with Tukey HSD test (*p* < 0.05). Data without significant differences are labeled with the same letters. (**c**) PR lengths of L*er* and *sprt2-1* mutants treated with spectinomycin and grown for 8d. Data are means ± SDs (n ≥ 11) Asterisks (*) indicate significant differences (*p* < 0.05, Student’s t-test).

Because the above results suggest that RFC3 is involved in plastid rRNA biosynthesis, we examined whether the *sprt2-1* mutation could counteract the failure of plastid ribosome function caused by genetic or pharmacological means. For this, we used *prps17-1* mutants, which are L*er* background mutants that lack the plastid ribosomal protein PRPS17 [45]. Similar to *rfc3-2* mutants, *prps17-*1 mutants form shorter roots than those in WT plants. On the other hand, we did not find clear visible phenotypes of *sprt2-1* single mutants in our experimental condition (Figure 6a). We generated *sprt2-1 prps17-1* mutants, which formed PRs of similar length to those of *prps17-1* mutants (Figure 6a,b). Treatment of WT plants with plastid translation inhibitors (e.g., spectinomycin) caused various phenotypes similar to those of *rfc3-2* mutants [35]. To determine whether the *sprt2-1* mutation could counteract the effects of spectinomycin on PR growth, *sprt2-1* mutants were grown on medium containing various concentrations of spectinomycin. In the presence of > 2 mg/L spectinomycin, both WT and *sprt2-1* mutant plants formed shorter roots, similar to those of *rfc3-2* mutants (Figure 6c). However, *sprt2-1* mutants showed greater sensitivity to 1 mg/L spectinomycin, compared to WT plants (Figure 6c). These findings indicated that the *sprt2-1* mutation does not restore plastid ribosome deficiency, and that RFC3 and SPRT2 have a specific functional relationship.

### 2.5. Identification of the sprt2-1 Mutation Site

**Figure 7:**
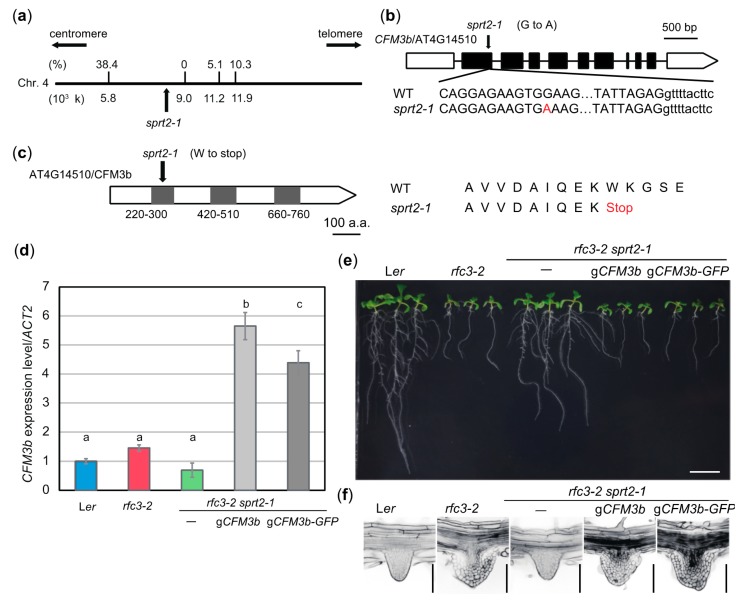
*SPRT2* corresponds to *CFM3b* that encodes a chloroplast RNA splicing and ribosome maturation (CRM)-domain protein. (**a**) Genetic mapping of the s*prt2-1* mutation site. A part of chromosome 4 is indicated by a bold line; nucleotide position is indicated below. Values on the bold line are recombination frequencies between single nucleotide polymorphisms and the *sprt2-1* mutation site. (**b**) Gene structure of *CFM3b* (At4g14510). The mutation point of *sprt2-1* is shown. (**c**) Protein structure of CFM3b. Gray boxes indicates CRM domains. The mutation point of *sprt2-1* is shown. (**d**) *CFM3b* expression levels determined by RT-qPCR. Data are means ± SDs. (n = 3). Statistical analysis was carried out by one-way ANOVA with Tukey HSD test (*p* < 0.05). Data without significant differences are labeled with the same letters. (**e**) Seedlings of *sprt2-1* complementation lines. Plants were grown on 1/2 MS medium containing 3% sucrose for 11 d. Bar = 1 cm. (**f**) LR phenotypes of the complementation lines. Bars = 100 μm.

**Figure 8:**
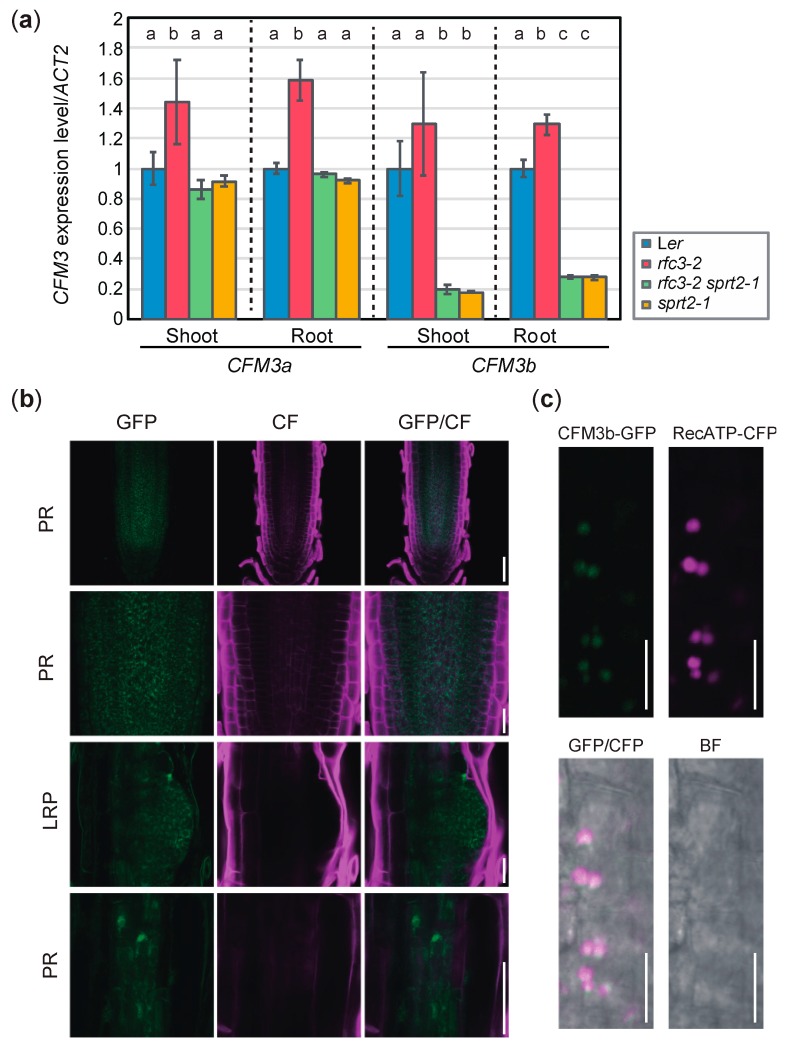
Expression level and pattern of *CFM3b*. (**a**) Expression levels of *CFM3a* and *CFM3b* in shoots and roots determined by RT-qPCR and normalized with *ACT2* expression level. Data are means ± SDs (n = 3). Statistical analysis was carried out by one-way ANOVA with Tukey HSD test (*p* < 0.05) separately for shoot and root samples. Data without significant differences are labeled with the same letters. (**b**) Expression patterns of p*CFM3b*::g*CFM3b-GFP* in root apical meristem (RAM) of PR and LRP of *rfc3-2 sprt2-1* mutants. Bars = 50 μm (top and bottom), 20 μm (middle). Cell wall was stained by calcofluor white (CF). (**c**). Colocalization analysis of CFM3b-GFP and RecATP-CFP in lateral root cap cells of PR. Bars = 10 μm.

To identify the causal gene in *sprt2-1* mutants, we initially attempted a classical map-based approach. It was necessary to obtain an *rfc3* allele in the Colombia (Col) background to map the chromosomal location of the *SPRT2* locus. We found two T-DNA insertion lines, *rfc3-3* and *rfc3-4*, both of which were likely null alleles because *RFC3* transcripts were almost undetectable (Figure S4a–c). However, *rfc3-3* and *rfc3-4* mutants produced normal LRs and shorter PRs (Figure S4d). F1 plants from a cross between *rfc3-2* and *rfc3-3* mutants produced normal LRs with short PRs (Figure S4d), suggesting a dominant modifier locus in the Col background. Because of this background effect, we abandoned this approach. We then carried out next-generation sequencing of *rfc3-2 sprt1-1* and *rfc3-2 sprt2-1* mutants. Extraction of *rfc3-2 sprt2-1*-specific polymorphisms, compared to those of *rfc3-2 sprt1-1* mutants, and their mapping on corresponding chromosomal positions suggested that four single nucleotide polymorphisms (SNPs) were localized in an approximately 500-kb region of chromosome 4 (Figure 7a). We crossed *rfc3-2 sprt2-1* mutants with *rfc3-2* mutants and selected double-homozygous plants from their F2 progenies. Using the four SNPs as molecular markers, we found tight linkages between each of these SNPs and the *SPRT2* locus (Figure 7a). Furthermore, one SNP showed complete linkage with the *sprt2-1* mutant phenotype; it created a nonsense mutation in At4G14510, which encodes CFM3b (Figure 7b, c). The defective protein was predicted to be truncated at the center of the first CRM domain (Figure 7b,c). RT-qPCR of *rfc3-2 sprt2-1* mutant showed that the *CFM3b* transcript levels tended to be slightly reduced, compared to the levels in WT plants; however, this difference was not statistically significant (Figure 7d). In another experiment, *CFM3b* expression was markedly reduced in *rfc3-2 sprt2-1* and *sprt2-1* mutants (Figure 8a), suggesting that the mutated *sprt2-1* transcripts underwent nonsense-mediated mRNA decay. CFM3b is a paralog of CFM3a that plays a role in the splicing of a subset of group IIB introns in plastids [23]. To examine whether *CFM3b* was the causal gene in *sprt2-1* mutants, we introduced a WT copy of a *CFM3b* genomic fragment, translationally fused or not to *GFP* cDNA (p*CFM3b*::g*CFM3b-GFP* or p*CFM3b*::g*CFM3b*), into *rfc3-2 sprt2-1* mutants. RT-qPCR confirmed that the expression of *CFM3b* was increased in these lines (Figure 7d). As expected, these transgenic lines produced shorter PRs with abnormal LRs, as observed in *rfc3-2* mutants (Figure 7e,f). Therefore, the results indicated that *SPRT2* corresponds to *CFM3b* (At4g14510). Sequencing of the *CFM3b* coding region in *rfc3-2 sprt1-1* and *rfc3-2 sprt3-1* mutants yielded no mutations.

### 2.6. Expression Pattern of CFM3b and Subcellular Localization of its Product

We examined expression levels of *CFM3a* and *CFM3b* in *rfc3-2*, *rfc3-2 sprt2-1*, and *sprt2-1* mutants. We noted that *CFM3a* exhibited elevated expression in *rfc3-2* mutant shoots and roots, compared to the shoots and roots of WT plants (Figure 8a). Similarly, *CFM3b* expression level in *rfc3-2* roots was slightly higher than the levels in WT roots although such a difference was not statistically significant in shoots (Figure 8a). In contrast, *CFM3a* expression levels in *rfc3-2 sprt2-1* and *sprt2-1* mutants were similar to those in WT plants (Figure 8a). Therefore, the findings indicated that *CFM3a* and *CFM3b* are positively regulated by a defect caused by the *rfc3-2* mutation. *CFM3b* expression levels in *rfc3-2 sprt2-1* and *sprt2-1* mutants were reduced compared those in WT plants (Figure 8a). We next examined the expression pattern of *CFM3b* and localization of the CFM3b protein using p*CFM3b*::g*CFM3b-GFP*/*rfc3-2 sprt2-1* plants. CFM3b-GFP was very weakly expressed throughout the roots (Figure 8b), suggesting that the CMF3b mRNA level was low and/or that CFM3b protein was unstable. At the subcellular level, CFM3b-GFP fluorescence was observed in the form of many small dots with structures in cells of the primary RAM and LRPs (Figure 8b). Colocalization analysis was performed by crossing a p*35S*::*RecATP-CFP* reporter [35] and a p*CFM3b*::*gCFM3b-GFP* in the *rfc3-2 sprt2-1* background. The fluorescence signals of CFP and GFP merged in cells of the RAM (Figure 8c). These results suggested that CFM3b was localized to root plastids. In a previous study, GFP fused to the transit peptide of CFM3b localized to plastids when transiently expressed in onion epidermal cells [23]; this finding is consistent with the above results.

### 2.7. RFC3 Interacts with CFM3b in Plastids

**Figure 9:**
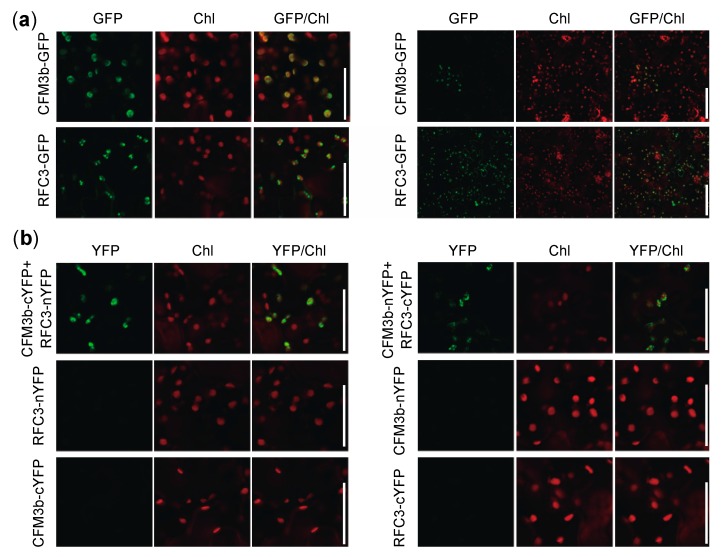
Cellular localization and bimolecular fluorescence complementation (BiFC) analysis of RFC3 and CFM3b. (**a**) RFC3 and CFM3b localization in plastids. RFC3-GFP and CFM3b-GFP were transiently expressed in *N. benthamiana* leaves. Bars in left and right panels indicate 50 and 100 μm, respectively. (**b**) BiFC analysis of RFC3 and CFM3b. Fluorescence resulting from complementation of the N-terminal part of YELLOW FLUORESCENT PROTEIN (YFP) fused with RFC3 (RFC3-nYFP) or CFM3b (CFM3b-nYFP) and the C-terminal part of YFP fused with CFM3b (CFM3b-cYFP) or RFC3 (RFC3-cYFP) were observed in epidermal cells of *N. benthamiana* leaves. Chl indicates chlorophyll autofluorescence. Bars = 50 μm.

Because both RFC3 and CFM3b localized to root plastids (Figure 8) [23,35], we analyzed whether RFC3 and CFM3b interacted with each other. We examined this possibility by yeast two-hybrid analysis and coimmunoprecipitation assays in *Nicotiana benthamiana* leaves. However, both attempts were unsuccessful; CFM3b-GFP was not detectable by Western blotting, even in input samples, despite expression of the fusion gene driven by the 35S promoter (data not shown). Next, RFC3-GFP and CFM3b-GFP were each transiently expressed in *N. benthamiana* leaves. A strong RFC3-GFP signal was observed in most pavement cells; however, a CFM3b-GFP signal was only observed occasionally (Figure 9a, right panel). Closer inspection indicated that both fusion proteins were localized in plastids (Figure 9a, left panel).

Next, we performed bimolecular fluorescence complementation (BiFC) analysis using *N. benthamiana* leaves. The N- or C-terminal part of a YELLOW FLUORESCENT PROTEIN (YFP) fragment (nYFP or cYFP) was fused to the C-terminal end of RFC3 and CFM3b; the fusion proteins were transiently expressed individually or in combination in *N. benthamiana* leaves. YFP fluorescence was reconstituted in plastids of epidermal cells when CFM3b-cYFP and RFC3-nYFP were coexpressed, as well as when CFM3b-nYFP and RFC3-cYFP were coexpressed (Figure 9b). No fluorescence signal was observed when each fusion protein was expressed alone (Figure 9b). These results suggested that RFC3 and CFM3b interact with each other in plastids.

## 3. Discussion

The molecular function of RFC3, a member of the bacterial RPS6 family, has been unclear because a *bona fide* PRPS6, which is phylogenetically related to cyanobacterial RPS6, is found in the 30S subunit [4,35,46]. Recently, a rice mutant, *thermo-sensitive chlorophyll-deficient mutant11* (*tcd11*) was shown to be defective in an RFC3 ortholog [47]. TCD11 was reported to be a plastid RP, but supporting biochemical evidence is lacking [47]. Interestingly, several ribosomal protein homologs act as ribosome biogenesis factors during cytosolic ribosome biogenesis in *Saccharomyces cerevisiae* (yeast) [48]. In addition, *rfc3* mutants had significantly reduced levels of 16S and 23S rRNAs (Figure 5) [35]. Based on these lines of circumstantial evidence, we hypothesized that RFC3 has an extra-ribosomal function, potentially in the biogenesis and/or stabilization of plastid rRNA. To gain insight into the molecular function of RFC3, we isolated suppressor mutants of the *rfc3-2* phenotype and found that *CFM3b* is the causal gene for the *sprt2* phenotype.

CFM3b is a paralog of the group II intron splicing factor CFM3a [23]. Because BiFC assays suggested that RFC3 and CFM3b interact in plastids and the *sprt2-1* mutation recovered the reduced plastid rRNA level in *rfc3-2* mutants, these two proteins likely directly or indirectly influence the rRNA level in plastids. Failure of the *sprt2-1* mutation to recover the spectinomycin- or *prps17* mutation-induced PR growth defect suggests that the *sprt2-1* mutation specifically affects a pathway in which RFC3 plays an important role in the maintenance of a sufficient plastid rRNA level. Therefore, it is feasible that nearly all aspects of *rfc3-2* mutant phenotypes were recovered by the *sprt2-1* mutation.

A number of mutants defective in plastid rRNA accumulation have been reported. These mutants can be classified into two groups based on the patterns of changes in rRNA levels. The first group is associated with specific or preferential reduction of rRNAs in the 30S or 50S subunit. For example, *Arabidopsis* mutants defective in a 30S ribosomal protein (i.e., *prps5*, *prps17*, and *plastid-specific ribosomal protein4* [*psrp4*]) [45,49,50], and a 50S ribosomal protein (i.e., *prpl6*/*embryo defective2394* [*emb2394*], *prpl24*/*suppressor of variegation8* [*svr8*], and *psrp5*) [45,50,51,52] exhibit reduced levels of 16S and 23S rRNAs, respectively. Similarly, mutants defective in some ribosome biogenesis factors show reduced levels of mature 16S rRNA (*rap* and *rbfa domain-containing protein1* [*rbf1*]) [53,54] and mature 23S rRNA (*svr7*) [55], respectively.

The second group shows general reductions in the levels of plastid rRNAs. This group includes a wide range of plastid proteins, as well as *rfc3* mutants. *prps21* is a viable mutant with a relatively strong defect in shoot growth and has reduced levels of 16S and 23S rRNAs [56]. Similarly, co-suppression lines of *PRPL15* in *Arabidopsis* and a knockout line of *prps15* in tobacco have shown reductions in 16S and 23S rRNA levels [57,58]. PALE CRESS (PAC) binds to 23S rRNA and promotes 50S subunit biogenesis, while the *pac* mutants dramatically reduce levels of both 16S and 23S rRNAs [59]. SUPPRESSOR OF THYLAKOID FORMATION1 (SOT1) binds the 5′ end of 23S and 4.5S rRNA precursors, whereas the *sot1* mutants reduce the levels of 23S, 4.5S, 16S, and 5S rRNAs [60]. In addition, mutants defective in translocon (*translocon at the inner envelope membrane of chloroplasts56* [*tic56*] and *plastid protein import2* [*ppi2*]) [61], transcription (*plastid transcriptionally active chromosome3* [*ptac3*]) [62], translation (*svr3* and *mitochondrial transcription termination factor6* [*mterf6*]) [63,64], splicing (*sot5*) [65], and RNA editing (*early chloroplast development1* [*ecd1*]) [66] show reduced levels of 16S and 23S rRNAs. These phenotypes are likely related to the inability to translate plastid-encoded ribosomal proteins. Indeed, treatment of WT plants with the plastid-translation inhibitors lincomycin [59] and spectinomycin [35] results in reduced levels of 16S and 23S rRNAs. In contrast, YbeY and RNR1 are involved in maturation of the termini of multiple rRNA species; mutations of these proteins result in markedly reduced levels of 16S and 23S rRNAs [67,68]. Therefore, reduced levels of 16S and 23S rRNAs can result from defects in (i) ribosome biogenesis factors broadly involved in 30S and 50S biogenesis; (ii) those specifically involved in 30S or 50S biogenesis; or (iii) plastid proteins not directly involved in ribosome biogenesis, although they secondary impair translation in plastids.

*rfc3* likely belongs to one of the above three categories. With regard to rRNA processing patterns, the levels of nearly all rRNA processing intermediates and mature 16S and 23S rRNAs in WT plants were markedly reduced in *rfc3* mutants; these defects were recovered by *sprt2* mutations. An exception was 3.0-kb precursors of 23S rRNA, which exhibited overaccumulation in *rfc3-2* mutants; this finding was indicative of the rate-limiting step of rRNA processing in *rfc3-2* mutants. Interestingly, *rfc3-2* mutants exhibited reduction of the 16S rRNA level to a greater degree than that of the 23S rRNA level, suggesting that RFC3 plays an additional role in the processing of 16S rRNA precursors. Notably, only *rfc3-2 sprt2-1* mutants exhibited overaccumulation of 1.7-kb 16S rRNA precursors, among all plant lines examined. If CFM3b affects rRNA levels in *rfc3-2* mutants, as a consequence of reduced plastid translation, *rfc3-2 sprt2-1* mutants should process 1.7-kb 16S rRNA intermediates normally; however, this phenomenon was not observed. These results suggest that (i) CFM3b likely affects one or more steps in rRNA processing or stability; (ii) RFC3 may prevent an unfavorable effect of CFM3b on rRNA processing; and (iii) RFC3 may also be involved in the processing of 1.7-kb 16S rRNA precursors.

Consistent with the results of an earlier report that did not found visible *cfm3b* phenotypes [23], *sprt2-1* single mutants also showed an almost normal rRNA processing pattern, suggesting that CFM3b plays only a minor role in WT plants. In contrast, *sprt2-1* mutants exhibited recovery of the reduction in rRNA levels observed in *rfc3-2* mutants. Therefore, RFC3 contributes to plastid rRNA biosynthesis through suppression of the non-specific actions of CFM3b. From a broader perspective, the role of CRM domain proteins in RNA metabolism requires specific RNA conformations. For example, CRS1 facilitates folding of the group II intron of *atpF* [16]. The RNA chaperone activity CFM4 complements the lack of such activity in an *E. coli* strain [27]. Furthermore, CFM3a plays distinct roles in plastids and mitochondria: It promotes group IIB intron splicing and is involved in mitochondrial 18S rRNA processing, respectively [23]. These examples suggest that CRM domains can interact with distinct RNA species with complex structures. In the absence of RFC3, CFM3b may directly or indirectly inhibit processing of rRNA. Because RFC3 and CFM3b interact with each other, RFC3 may sequester CFM3b to prevent its interactions with non-client RNAs. In such a case, RFC3 has a role to ensure the accuracy of RNA metabolism in plastids. A second possibility is that a potential defect in group IIB intron splicing by the *sprt2-1* mutation promotes rRNA processing and/or stability. Among the suggested targets of CFM3 [23], *ndhB*, *petB*, and *petD* encodes proteins involved in photosynthetic electron transport and therefore do not have a direct link with rRNA biogenesis. The remaining targets of CFM3 encode ribosomal proteins (*rpl16* and *rps16*) and a tRNA (*trnG*). If their splicing is impaired, rRNA biogenesis is expected to be negatively affected, but this is the opposite of what we observed and therefore this possibility is unlikely.

There are several examples of mutations or genetic manipulations that suppress the phenotypes caused by defects in ribosome biogenesis. For example, in yeast 40S ribosome biogenesis, depletion of Rio1 inhibits 20S rRNA cleavage and results in a lethal phenotype; however, this can be bypassed by missense mutations of Nob1 and Pno1 [69]. Similarly, the lethal phenotype of a mutant defective in yeast 60S ribosome biogenesis factor Nsa1 can be suppressed by mutations in Mak5, Nop1, and Nop4 [70]. These and other studies reveal the existence of multiple checkpoints in ribosome assembly pathways [69,71]. A pioneering work showed that the expression of a dominant-negative form of ribosome biogenesis factor Bop1 inhibits ribosome biogenesis and causes cell cycle arrest in cultured mouse cells; this cell cycle arrest could be avoided by inactivation of p53 [72]. A number of subsequent works established the ribosome stress response pathway, in which defects in ribosome biogenesis induce an interaction between RPs and the E3 ubiquitin ligase MDM2, then stabilize p53, a master regulator of the stress response; this interaction induces cell cycle arrest [73]. Although plants do not have homologs of MDM2 or p53, a ribosome stress response pathway is present in *Arabidopsis* [74,75]. A suppressor screening of a ribosome biogenesis mutant, *root initiation defective2* (*rid2*), showed that *suppressor of rid two1* (*sriw1*) disrupted the NAC transcription factor gene *ANAC082* [74]. In *rid2 sriw1* mutants, the growth defect of the *rid2* phenotype was suppressed by the *sriw1* mutation, without recovery of defective ribosome biogenesis [74]. Therefore, SRIW1 might share some common attributes with the animal p53 with regard to ribosome stress response pathway.

Given that suppressors of ribosome biogenesis mutants yielded important biological findings, the correlation between the plastid rRNA level and the LR developmental phenotype in *rfc3-2* and *rfc3-2 sprt2-1* mutants is noteworthy. This correlation suggests that impaired plastid rRNA biogenesis alters the expression patterns of nuclear-encoded genes involved in LR development. It remains unclear whether these changes are secondary to the general growth defect of the *rfc*3 phenotype or represent a specific outcome of “plastid ribosome stress”. The plastid-to-nucleus signaling pathways, termed retrograde signaling, have been extensively studied with a focus on chloroplast development and regulation of photosynthesis [76]. GENOMES UNCOUPLED1 (GUN1) is considered an integrator of signals in response to perturbations in the redox state, tetrapyrrole biosynthesis, and plastid gene expression [77]. Lincomycin is used to impair plastid gene expression and evoke GUN1-dependent retrograde signaling [77]. Interestingly, *gun1* synergistically enhances the shoot growth defect when combined with *prpl11*, but not *prps21* or *prps1*; moreover, GUN1 physically interacts with several RPs in plastids [56]. Concerning development, the leaf adaxial-abaxial boundary is destabilized by lincomycin treatment or by *enhanced fil expression domain2* (*enf2*) mutations, which disrupt a plastid-localized PotD homolog [78]. Such destabilization of adaxal-abaxial boundary occurs in a GUN1-dependent manner [78]. In contrast, it is unknown whether GUN1 has a role in roots. The relationships among *rfc3*, *sprt2*, and *gun1* phenotypes are of particular interest and will be examined in a future study.

In summary, the bRPS6-family member RFC3 likely contributes to plastid rRNA biogenesis by preventing nonspecific effects of CFM3b, thereby enhancing the accuracy of RNA metabolism in plastids. In addition, the normal function of plastids is related to the development of LRs. The relationship between plastid function and LR formation will be elucidated by further biochemical analysis of functional relationships between RFC3 and CFM3b, as well as identification of the responsible genes in other *sprt* mutants.

## 4. Materials and Methods 

### 4.1. Plant Materials and Growth Conditions

WT plants used in this study were L*er* and Col-0. Alleles of *rfc3* were described previously (*rfc3-2*) [34] or obtained from the Arabidopsis Biological Resource Center (*rfc3-3* [Salk_015990] and *rfc3-4* [Sail_557_D02]). p*WOX5*::*GFP* [79], p*PLT3*::*CFP* [44,80], *QC25*::*GUS* and *QC184*::*GUS* [81], p*35S*::*RecATP-CFP* [35], and *prps17-1* [45] were reported previously. The reporter lines or mutants were introgressed into the L*er* background by at least four successive genetic crosses. Plants were cultured under long-day conditions (16 h light, 8 h dark) at 22 °C. The light intensity was 50 or 20 μmol m^−2^ s^−1^ when rock wool or solid medium was used, respectively. Half-strength Murashige and Skoog (MS) medium [82] supplemented with 3% or 2% (w/v) sucrose and solidified with 0.5% (w/v) gellan gum was used to observe root phenotypes. Spectinomycin (Wako Pure Chemical, Osaka, Japan) was added to MS medium at final concentrations up to 6 mg/L as a plastid ribosome inhibitor. Plants were grown on rock wool covered with powdered peat moss to observe shoot phenotypes or to generate transgenic plants. *N. benthamiana* was grown on peat containing nutrients (Sakatanotane Co., Yokohama, Japan) and *Arabidopsis* was grown with 0.5 g/L Hyponex (Hyponex Japan, Osaka, Japan) supplied daily as fertilizer.

### 4.2. Isolation of Suppressor Mutants of rfc3-2

Mutagenesis was carried out by incubating approximately 10,000 *rfc3-2* seeds in 0.1 to 0.3% (v/v) ethyl methanesulfonate solution for 16 h. Three suppressor lines were identified by visual inspection of LRs in approximately 10,000 M2 seedlings. These suppressors were crossed with *rfc3-2* mutants three times before use in experiments.

### 4.3. Genetic Mapping of the sprt2-1 Mutation Site

Genomic DNA was extracted for resequencing of *rfc3-2 sprt1-1* and *rfc3-2 sprt2-1* mutants and for genetic mapping of the *sprt2-1* mutation site according to [83] with an additional extraction step with phenol:chloroform:isoamylalcohol (25:24:1). The genomic DNA of *rfc3-2 sprt1-1* and *rfc3-2 sprt2-1* mutants was subjected to paired-end sequencing on a HiSeq 2500 (Illumina, San Diego, CA, USA) at Hokkaido System Science Co., Ltd. (Sapporo, Japan). G to A and C to T transversions were identified and *sprt2-1*-specific changes were recovered. *rfc3-2 sprt2-1* segregants in an F2 population, obtained from a cross between *rfc3-2 sprt2-1* and *rfc3-2* mutants, were used for genetic mapping. The mutation point of *sprt2-1* was localized using the polymorphisms between *rfc3-2 sprt2-1* and *rfc3-2* mutants as genetic markers.

### 4.4. Complementation Test of sprt2

*CFM3b* genomic fragments containing an approximately 1.4-kb promoter region and the transcribed region, with or without the termination codon, were amplified by polymerase chain reaction (PCR) and cloned into pENTR/D-TOPO (Thermo Fisher Scientific, Rockford, IL, USA). Next, the *CFM3b* sequences were transferred into the binary vectors pBG [84] and pHWG [85], respectively, using Gateway LR clonase II (Thermo Fisher Scientific, Walham, MA, USA). *rfc3-2 sprt2-1* mutant plants were transformed with these vectors by the floral-dip method [86]. Homozygous plants with a single T-DNA insertion were used in experiments.

### 4.5. Microscopy

Structure of LRPs was observed in detail by the pseudo-Schiff-propidium iodide (mPS-PI) method, as described previously with some modifications [35,87]. LRPs were observed using an LSM 800 microscope (Carl Zeiss, Zena, Germany). Roots of *QC25*::*GUS* and *QC184*::*GUS* lines were submerged in ice-cooled 90% (v/v) acetone for fixation, then incubated in β-glucuronidase (GUS) reaction solution (500 μg/mL 5-bromo-4-chloro-3-indolyl-β-D-glucuronic acid, 100 mM NaPO_4_ [pH 7], 3 mM potassium ferricyanide, 10 mM EDTA, 0.1% [v/v] Triton X-100) overnight at 37 °C for GUS staining. The roots were cleared with chloral hydrate solution [84] and observed under a DM2500 microscope equipped with a DFC420C camera (Leica Microsystems, Wetzlar, Germany). Plants were grown in 1/2 MS medium containing 3% (w/v) sucrose for 3 weeks and chlorophyll autofluorescence was observed using an LSM 800 microscope. p*WOX5*::*GFP*, p*PLT3*::*CFP*, p*35S*::*RecATP-CFP* lines stained with or without 10 μg/mL propidium iodide (PI) were observed by LSM800. p*CFM3b*::g*CFM3b-GFP* line was fixed, stained with Calcofluor White and cleared according to [88], and observed by LSM800.

### 4.6. Transient Assays

Transient assays in *N. benthamiana* were carried out according to [89,90]. *RFC3* and *CFM3b* cDNAs without the termination codon were cloned into pENTR/D TOPO, using the primers in Table S1. Next, the inserts were transferred into pB4GWnY, pB4GWcY, or pH35WG vectors carrying N- and C-terminal parts of YFP or full-length GFP, respectively, by LR clonase [35,91]. These constructs were introduced into *Agrobacterium tumefaciens* C58C1 by electroporation. *A. tumefaciens* were cultured, collected by centrifugation, diluted with dH_2_O to an A_600_ of 0.8, and mixed with acetosyringone (final concentration of 150 μM). The *A. tumefaciens* solution was infiltrated into 4–5-week-old leaves of *N. benthamiana* using a syringe without a needle. *N. benthamiana* plants were grown for 2–3 days and the leaves were harvested. Leaf mesophyll and epidermal cells were observed by LSM800.

### 4.7. Northern Blot Analysis of Plastid rRNAs

Total RNAs were extracted from roots of 8-day-old seedlings grown on half-strength MS agar supplemented with 3% (w/v) sucrose using TRI regent (Merck, Darmstadt, Germany) according to the manufacturer’s instructions. Total RNA was separated on a 2% (w/v) agarose gel containing formaldehyde and transferred to a positively charged nylon membrane (Merck) by capillary transfer. Template DNAs for in vitro transcription were amplified by PCR using specific primers (Table S1) and DIG-labeled RNA probes were synthesized using the DIG Northern Starter Kit or DIG RNA Labeling Kit (Roche, Basel, Switzerland). The membranes were hybridized with DIG-labeled RNA probes and washed; hybridization signals were detected using an ImageQuant LAS 4000mini instrument (GE Healthcare, Chicago, IL, USA), in accordance with the manufacturer’s instructions.

### 4.8. RT-qPCR

Seedlings grown on half-strength MS agar supplemented with 3% (w/v) sucrose for 11 d and whole seedlings, shoots, or roots were frozen in liquid nitrogen; total RNA was extracted with TRI reagent (Merck), in accordance with the manufacturer’s instructions. After DNase I treatment, 2 μg of total RNA were subjected to RT-PCR using the SuperScript III First-Strand System (Thermo Fisher Scientific). Oligo (dT) primers were used to amplify mRNAs from nuclear-encoded genes; random hexamers were used to amplify mRNAs from plastid-encoded gene and cytoplasmic rRNAs. Quantitative PCR was performed with Go Taq qPCR Master Mix (Promega, Madison, WI, USA) using the QuantStudio™ 12K Flex Real-Time PCR System (Thermo Fisher Scientific). The expression levels of nuclear- and plastid-encoded genes were calculated by the ΔΔCT method [92] and normalized to *ACT2* and 18S rRNA, respectively. Primers are listed in Table S1. 

## Supplementary Materials

The following are available online at https://www.mdpi.com/2223-7747/9/3/328/s1, Figure S1: Root phenotypes of homozygous and heterozygous *sprt* mutants grown on a half-strength of MS medium supplemented with 2% (w/v) sucrose, Figure S2: Expression of p*WOX5*::*GFP* in later-stage LRs, Figure S3: Expression of QC184 marker, Figure S4: Effect of genetic background on lateral root phenotype, according to *rfc3* alleles, Table S1: Information of primers used in this study.
