# Supplementary material for: The bRPS6-Family Protein RFC3 Prevents Interference by the Splicing Factor CFM3b during Plastid rRNA Biogenesis in Arabidopsis thaliana
Source: Plants (Basel). 2020 Mar 4;9(3):328. doi: 10.3390/plants9030328 (PMC7154815; doi:10.3390/plants9030328)
Supplement: Supplementary file 1 [file plants-09-00328-s001.zip › Figure S1 (revision).pdf]

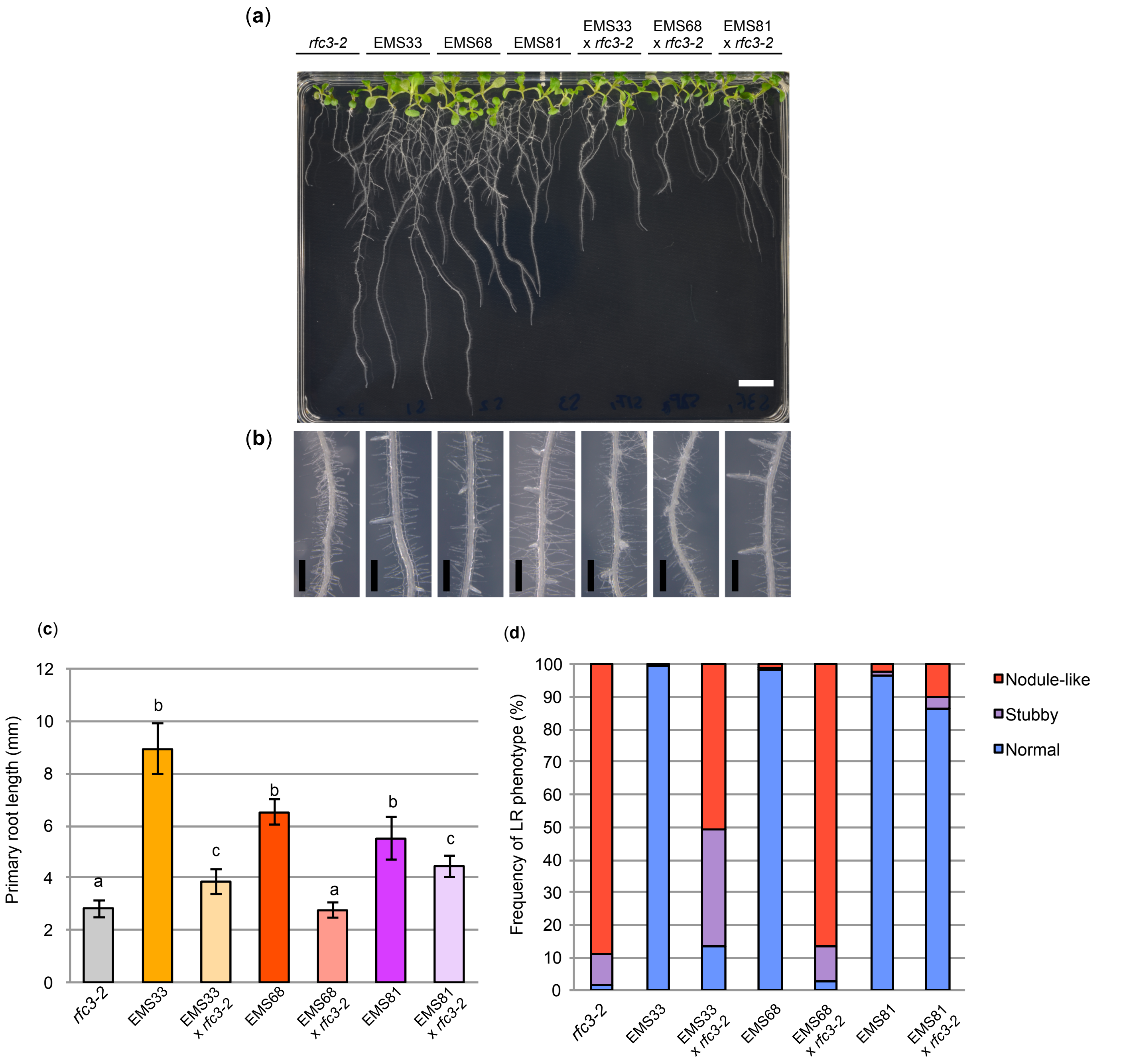

Figure S1. Root phenotypes of homozygous and heterozygous suppressor mutants grown on a half-strength of MS medium supplemented with 2% (w/v) sucrose. (**a**) Eleven-day-old seedlings. (**b**) Lateral roots. (**c**) Primary root lengths. Data are means ± SDs. (n = 20–24). Statistical analysis was carried out by one-way ANOVA with Tukey HSD test ($P < 0.05$) among *rfc3-2* mutants and the respective homozygous and heterozygous suppressor mutants. Data without significant differences are labeled with the same letters. (d) Frequency distributions of nodule-like, stubby, and normal lateral roots in each genotype (n = 423–822). Bars in (a) and (b) = 1 cm and 1 mm, respectively.
