# Supplementary material for: The bRPS6-Family Protein RFC3 Prevents Interference by the Splicing Factor CFM3b during Plastid rRNA Biogenesis in Arabidopsis thaliana
Source: Plants (Basel). 2020 Mar 4;9(3):328. doi: 10.3390/plants9030328 (PMC7154815; doi:10.3390/plants9030328)
Supplement: Supplementary file 1 [file plants-09-00328-s001.zip › Figure S2.pdf]

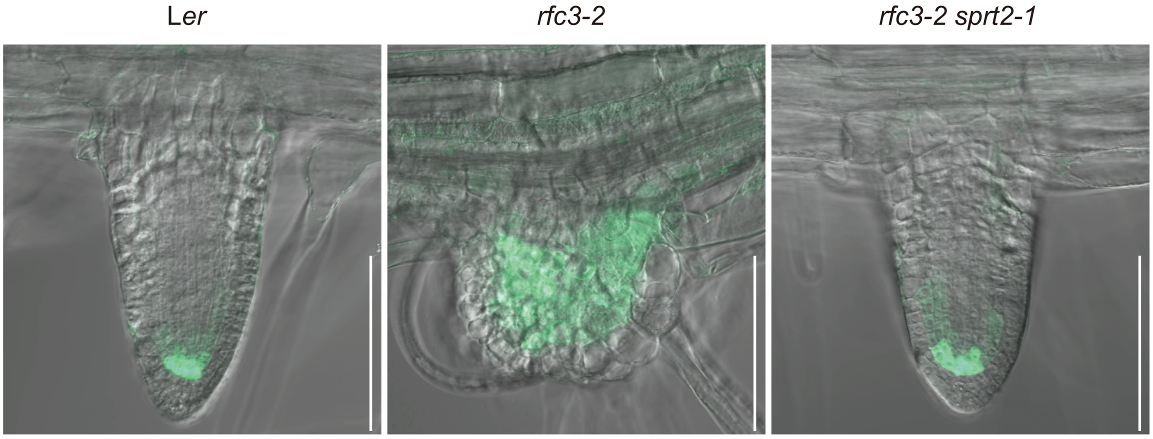

Figure S2. Expression of p*WOX5*::*GFP* in later-stage LRs. p*WOX5*::*GFP* patterns (green) merged with bright-field (BF) images in LRs. Bars = 100 µm.
