# Supplementary material for: The bRPS6-Family Protein RFC3 Prevents Interference by the Splicing Factor CFM3b during Plastid rRNA Biogenesis in Arabidopsis thaliana
Source: Plants (Basel). 2020 Mar 4;9(3):328. doi: 10.3390/plants9030328 (PMC7154815; doi:10.3390/plants9030328)
Supplement: Supplementary file 1 [file plants-09-00328-s001.zip › Figure S3.pdf]

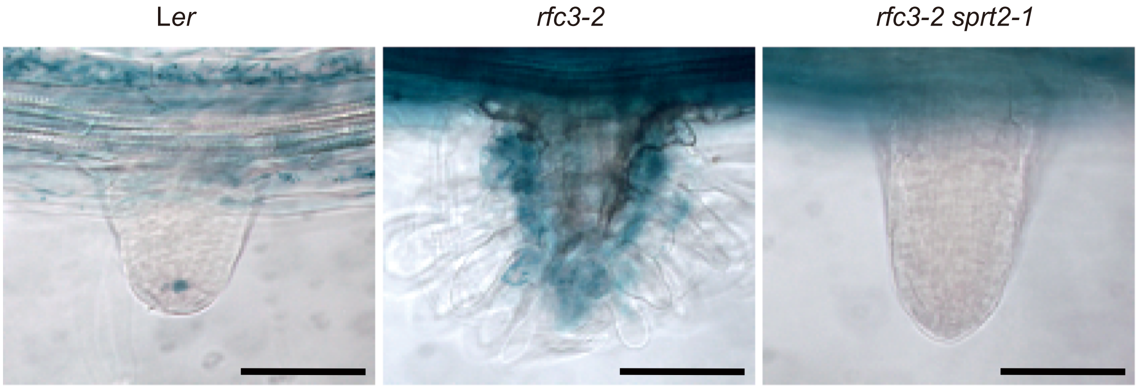

Figure S3. Expression of QC184 marker. LRs of the *QC184*::*GUS* lines in L*er*, *rfc3-2*, and *rfc3-2 sprt2-1* mutant backgrounds were stained with GUS. Bars = 50 μm.
