# Supplementary material for: The bRPS6-Family Protein RFC3 Prevents Interference by the Splicing Factor CFM3b during Plastid rRNA Biogenesis in Arabidopsis thaliana
Source: Plants (Basel). 2020 Mar 4;9(3):328. doi: 10.3390/plants9030328 (PMC7154815; doi:10.3390/plants9030328)
Supplement: Supplementary file 1 [file plants-09-00328-s001.zip › Figure S4.pdf]

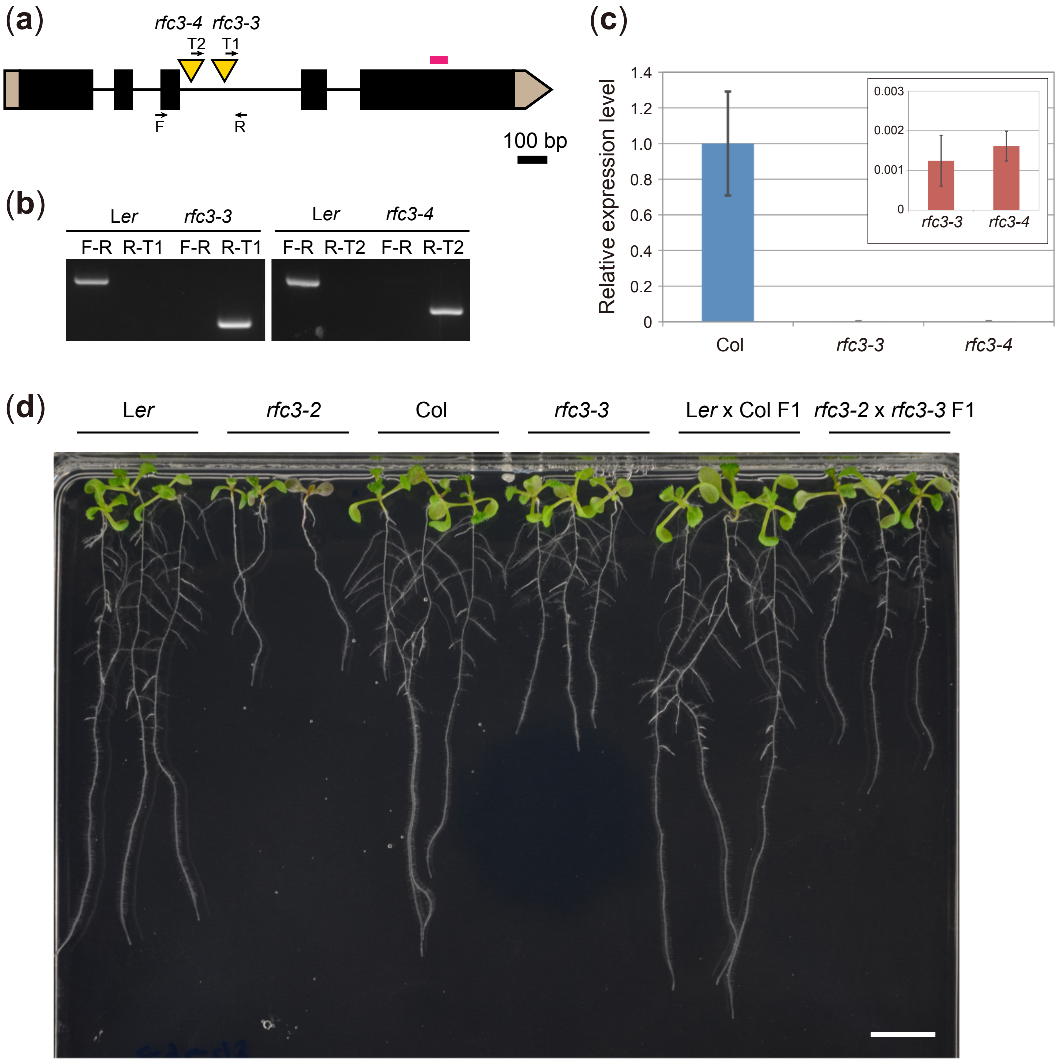

Figure S4. Effect of genetic background on lateral root phenotype, according to *rfc3* alleles. (**a**) T-DNA insertion alleles of *rfc3* mutants. T-DNA insertions are indicated by triangles. Primers used for the genotyping of *rfc3* alleles are indicated by arrows. Magenta line indicates the region amplified by RT-qPCR in (**c**). (**b**) Genotyping of *rfc3-3* and *rfc3-4* mutants. (**c**) Expression levels of *RFC3* in *rfc3* mutants, as determined by RT-qPCR (n = 3, means ± SDs). (**d**) Root phenotypes of *rfc3-2* mutant x *rfc3-3* mutant F1 plants grown on half-strength MS medium supplemented with 2% (w/v) sucrose for 11 d. Bar, 1 cm. Note defective and normal LR development in *rfc3-2* and *rfc3-3* mutants, respectively. LRs in *rfc3-2*/*rfc3-3* transheterozygous mutants were produced normally.
